# Short-term changes in klotho and FGF23 in heart failure with reduced ejection fraction—a substudy of the DAPA-VO_2_ study

**DOI:** 10.3389/fcvm.2023.1242108

**Published:** 2023-08-25

**Authors:** Carmen Mora-Fernández, Adora Pérez, Anna Mollar, Patricia Palau, Martina Amiguet, Rafael de la Espriella, Juan Sanchis, Jose Luis Górriz, María José Soler, Juan F. Navarro-González, Julio Núñez, DAPA-VO2 Investigators, Patricia Palau

**Affiliations:** ^1^ Research Unit, Hospital Universitario Nuestra Señora de Candelaria, Santa Cruz de Tenerife, Spain; ^2^ Cardiology Department, Hospital Clínico Universitario de Valencia, Universitat de València, INCLIVA, Valencia, Spain; ^3^ CIBER Cardiovascular, Madrid, Spain; ^4^ Cardiology Department, Hospital General de Castellón, FISABIO, Castellón, Spain; ^5^ Nephrology Department, Hospital Clínico Universitario de Valencia, Universitat de València, INCLIVA, Valencia, Spain; ^6^ Nephrology Department, Vall d’Hebron Barcelona Hospital Campus and Vall d’Hebron Research Institute (VHIR), Barcelona, Spain; ^7^ Nephrology Service, Hospital Universitario Nuestra Señora de Candelaria, Santa Cruz de Tenerife, Spain

**Keywords:** dapagliflozin, fibroblast growth factor 23, functional capacity, heart failure with reduced ejection fraction, klotho, peak oxygen consumption

## Abstract

The klotho and fibroblast growth factor 23 (FGF-23) pathway is implicated in cardiovascular pathophysiology. This substudy aimed to assess the changes in klotho and FGF-23 levels 1-month after dapagliflozin in patients with stable heart failure and reduced ejection fraction (HFrEF). The study included 29 patients (32.2% of the total), with 14 assigned to the placebo group and 15 to the dapagliflozin, as part of the double-blind, randomized clinical trial [DAPA-VO_2_ (NCT04197635)]. Blood samples were collected at baseline and after 30 days, and Klotho and FGF-23 levels were measured using ELISA Kits. Between-treatment changes (raw data) were analyzed by using the Mann-Whitney test and expressed as median (p25%–p75%). Linear regression models were utilized to analyze changes in the logarithm (log) of klotho and FGF-23. The median age was 68.3 years (60.8–72.1), with 79.3% male and 81.5% classified as NYHA II. The baseline medians of left ventricular ejection fraction, glomerular filtration rate, NT-proBNP, klotho, and FGF-23 were 35.8% (30.5–37.8), 67.4 ml/min/1.73 m^2^ (50.7–82.8), 1,285 pg/ml (898–2,305), 623.4 pg/ml (533.5–736.6), and 72.6 RU/ml (62.6–96.1), respectively. The baseline mean peak oxygen uptake was 13.1 ± 4.0 ml/kg/min. Compared to placebo, patients on dapagliflozin showed a significant median increase of klotho [Δ+29.5, (12.9–37.2); *p* = 0.009] and a non-significant decrease of FGF-23 [Δ−4.6, (−1.7 to −5.4); *p* = 0.051]. A significant increase in log-klotho (*p* = 0.011) and a decrease in log-FGF-23 (*p* = 0.040) were found in the inferential analysis. In conclusion, in patients with stable HFrEF, dapagliflozin led to a short-term increase in klotho and a decrease in FGF-23.

## Introduction

The mechanisms behind the cardiovascular and renal benefits of sodium-glucose cotransporter 2 inhibitors (SGLT2i) remain multiple and not fully clarified (1). Klotho-Fibroblast Growth Factor 23 (FGF-23) pathway is involved in the pathophysiology of cardiovascular and kidney complications (2). The effect of SGLT2i on this pathway, although suggested, remains mostly unknown. Given the cardio and nephroprotective effects of SGLT2i, we postulate dapagliflozin may exert changes in klotho/FGF-23 axis.

In this sub-study of a double-blind, randomized clinical trial (DAPA-VO_2_) (3), we aimed to test between-treatment changes (dapagliflozin vs. placebo) in klotho, FGF-23, and the ratio klotho/FGF-23 at 1-month following randomization. Additionally, we evaluated whether baseline values of klotho and FGF-23 were associated with 1-month changes in peak oxygen consumption (peak VO_2_) in stable outpatients with heart failure with reduced ejection fraction (HFrEF).

## Methods

### Study sample and procedures

This is a *post hoc* analysis of the DAPA-VO_2_ trial. It was an investigator-initiated, multicenter, double-blind, randomized clinical trial designed to evaluate the effect of dapagliflozin on 1 and 3-month peak-VO_2_ in patients with HFrEF. The patients were randomized 1:1 to receive either oral dapagliflozin 10 mg/daily or a matching placebo/daily [clinicaltrials.gov (NCT04197635)] in 3 institutions in Spain (Hospital Clinic Universitario de Valencia-Valencia, Hospital de Denia-Alicante, and Hospital Universitario Virgen de la Victoria-Malaga). Astra Zeneca supplied dapagliflozin and the matching placebo (similar characteristics as possible to the group receiving the active treatment regarding appearance, taste, and composition beyond the active drug).

Inclusion criteria included: (a) adult patients >18 years old with stable symptomatic heart failure with a NYHA II-III during the last 2-month; (b) left ventricular ejection fraction (LVEF) ≤40% documented in the last 3 months by echocardiography or cardiac magnetic resonance; (c) N-terminal pro-brain natriuretic peptide (NT-proBNP) ≥600 pg/ml; (d) estimated glomerular filtration rate (eGFR) ≥30 ml/min/1.73 m^2^ at enrolment; and (e) optimal and stable background treatment for HFrEF. Exclusion criteria were (a) inability to perform a valid (respiratory exchange ratio -RER- ≥1.05) baseline cardiopulmonary exercise test (CPET); (b) heart failure due to restrictive cardiomyopathy, active myocarditis, constrictive pericarditis, hypertrophic (obstructive) cardiomyopathy, or uncorrected severe primary cardiac valve disease; (c) myocardial infarction, unstable angina, stroke, or transient ischemic attack within 3 months prior to enrolment; (d) patients receiving therapy with an SGLT2i within 8 weeks prior to enrolment, or previous intolerance of an SGLT2i; (e) type 1 diabetes; (f) coronary revascularization (percutaneous coronary intervention or coronary artery bypass grafting) or cardiac valve repair/replacement within 12 weeks prior to enrolment, or planned to undergo any of these operations after randomization; (g) implantation of a cardiac resynchronization therapy (CRT) device within 12 weeks prior to enrolment or intent to implant a CRT device; (h) previous cardiac transplantation or implantation of a ventricular assistance device, or implantation expected after randomization; (i) symptomatic bradycardia or second or third-degree heart block without a pacemaker; (j) renal dysfunction (eGFR < 30 ml/min/1.73 m^2^) or prior admission for acute renal failure in the last 4 weeks; (k) pregnant or lactating women; (l) woman of childbearing age, unless they are using highly effective contraceptive methods; and (m) patients with severe hepatic impairment (Child-Pugh class C).

The study protocol was approved by Agencia Española del Medicamento y Productos sanitarios (AEMPS) and by Comité Ético de Investigación Clínica (CEIC) del Hospital Clínico Universitario de Valencia. Informed consent was obtained from each patient, and the study protocol conforms to the ethical guidelines of the 1975 Declaration of Helsinki as reflected in *a priori* approval by the institution's human research committee. LVEF was estimated by 2D-echocardiography using the Simpson Biplane method, and the blood pressure measurement technique was oscillatory. Blood samples were obtained the same day before the CPET and 30 days after. This substudy included 29 of 90 patients (32.2% of the total sample) in which frozen samples were available. Soluble Klotho was measured by a solid phase sandwich ELISA using the human soluble α-Klotho assay kit (Immuno-Biological Laboratories, Takasaki, Japan), with a sensitivity of 6.15 pg/ml (lower limit of quantification), and intra- and inter-assay coefficients of variation <3.1% and 6.9%, respectively. Intact FGF-23 levels were determined by Human FGF-23 ELISA Kit (EMD Millipore Corporation, Milford, MA, USA), with a sensitivity of 3.5 pg/ml (lower limit of quantification) and intra-and inter-assay coefficients of 9.5% and 6.85%, respectively. The values of both biomarkers were blinded to those researchers in charge of performing the CPET. N-terminal pro-brain natriuretic peptide (NT-proBNP) was measured using commercial enzyme immune analysis (Roche Elecsys® NT-proBNP), and eGFR was calculated using CKD-EPI equation.

### Statistical analysis

All statistical comparisons were made under a modified intention-to-treat principle. Continuous variables are expressed as medians [interquartile range (IQR)], and discrete variables are presented as percentages. At baseline, the means, medians, and frequencies among treatment groups were compared using the *T*-test, Mann-Whitney and chi-square tests, respectively. Assuming an alpha level of 0.05 (two-tailed test), we performed the power analysis to estimate the statistical power for detecting the observed effect size. Using the G*Power software. Within-group comparisons (pre-post) were evaluated by the Wilcoxon test. Observed between-treatment changes in klotho, FGF-23, and the ratio klotho/FGF-23 were tested by the Mann-Whitney test and expressed as median (percentile 25% to percentile 75%). We used a linear regression model to analyze changes in both biomarkers. All analyses included the baseline value of the biomarker as a covariate (ANCOVA framework) and potential confounders (age, sex, and estimated glomerular filtration rate). Klotho, FGF-23, and their ratio were also transformed to their natural logarithm to make their distributions more parametrical. The association between baseline values of klotho and FGF-23 and 1-month between-treatment changes in peak VO_2_ were also evaluated by linear regression analysis adjusted for the baseline value of peak VO_2_. Inferential estimates are presented as least square means with 95% confidence intervals (CIs) and *p*-values. All analyses were performed with STATA 16.1 [Stata Statistical Software, Release 16 (2019); StataCorp LP, College Station, TX, USA].

## Results

Klotho and FGF-23 were available in 29 patients (15 on dapagliflozin and 14 on placebo). The median (percentile 25% to percentile 75%) age of this sample was 68.3 (60.8–72.1), 23 (79.3%) were men. Most of them showed prior dyslipidemia (82.8%), hypertension (75.9%), were on stable NYHA class II (81.55), and were treated with guideline medical therapy. About half of the sample showed ischemic heart disease (58.6%) and atrial fibrillation (55.2%) of admission. About a third of patients displayed type 2 diabetes (31.0%). At baseline, the medians (percentile 25% to percentile 75%) of LVEF, eGFR, NT-proBNP, hemoglobin, klotho, and FGF-23 were 35.8% (30.5–37.8), 67.4 ml/min/1.73 m^2^ (50.7–82.8), 1,285 pg/ml (898–2,305), 14.2 (13–15.6), 623.4 pg/ml (533.5–736.6), and 72.6 RU/ml (62.6–96.1), respectively. GFR < 60 ml/min/1.73 m^2^ was present in 41.4% of the sample. At baseline, the mean peak VO_2_ was 13.1 ± 4.0 ml/kg/min. Baseline characteristics were not significantly different across both treatment arms, including the values of klotho and FGF-23 (Table 1).

**Table 1:** Baseline characteristics of the patients stratified by randomization arm.

Variables	All patients	Placebo	Dapagliflozin	*p*-value
*n*	29	14	15	
Demographic and medical history				
Age, years	68.3 (60.8–72.1)	65.3 (60.1–71.9)	70.9 (64.3–74.1)	0.667
Men, *n* (%)	23 (79.3)	11 (78.6)	12 (80.0)	0.924
Hypertension, *n* (%)	22 (75.9)	11 (78.6)	11 (73.3)	0.742
Diabetes mellitus, *n* (%)	9 (31.0)	3 (21.4)	6 (40)	0.280
Dyslipidemia, *n* (%)	24 (82.8)	11 (78.6)	13 (86.7)	0.564
Current smoker, *n* (%)	8 (27.6)	4 (28.6)	4 (26.7)	0.909
Prior history of IHD, *n* (%)	17 (58.6)	6 (42.9)	11 (73.3)	0.096
Prior history of COPD, *n* (%)	7 (24.1)	5 (35.7)	2 (13.3)	0.159
NYHA II, *n* (%)	22 (81.5)	11 (84.6)	11 (78.6)	0.684
Vital signs and EKG				
Heart rate, bpm	68.0 (60.5–77.0)	70.5 (65.0–81.0)	63.0 (58.0–73.0)	0.048
Systolic blood pressure, mmHg	119.0 (110.0–126.0)	110.0 (105.0–130.0)	120.0 (110.0–122.0)	0.684
Diastolic blood pressure, mmHg	60 (60–60)	60 (60–60)	60 (60–62)	0.784
Atrial fibrillation, (%)	16 (55.2)	10 (71.4)	6 (40.0)	0.089
Left bundle branch block, *n* (%)	4 (13.8)	3 (21.4)	4 (13.8)	0.249
Laboratory values				
Haemoglobin, g/dl	14.2 (13–15.6)	14.5 (13.0–15.6)	14.1 (12.5–15.7)	0.456
eGFR, ml/min/1.73 m^2^	67.4 (50.7–82.8)	70.3 (53.8–86.7)	65.7 (44.9–81.6)	0.456
eGFR < 60, ml/min/1.73 m^2^, *n* (%)	12 (41.4)	5 (35.7)	7 (46.7)	0.550
Serum sodium, mEq/L	139 (137–141)	138.5 (137–140)	139 (137–141)	0.235
NT-proBNP, pg/ml	1,285 (898–2,305)	1,251.5 (995–2,305)	1,285 (895–2,305)	0.964
CA125, U/ml	9 (6–16)	11 (8–17)	8 (6–16)	0.580
Klotho, pg/ml	623.4 (533.5–736.6)	584.8 (504.1–716.9)	684.2 (570.6–737.1)	0.283
FGF-23, RU/ml	72.6 (62.6–96.1)	70.8 (50.2–96.7)	73.5 (66.3–95.4)	0.874
Logarithm klotho	6.4 ± 0.2	6.4 ± 0.2	6.5 ± 0.2	0.296
Logarithm FGF-23	4.3 ± 0.4	4.3 ± 0.4	4.3 ± 0.4	0.759
Echocardiography				
LVEF, %	35.8 (30.5–37.8)	35.5 (30.5–38.9)	35.9 (30.4–37.8)	0.755
E/E’ ratio	12.9 (10.5–15.6)	13.2 (10.0–14.7)	12.5 (11.3–15.7)	0.496
CPET				
PeakVO_2_, ml/kg/min	13.1 ± 4.0	12.3 ± 3.1	13.9 ± 4.7	0.297
Percent predicted peakVO_2_, %	52.7 (48.1–63.3)	52.6 (47.9–62.8)	53.8 (50.2–65.6)	0.379
VE/VCO_2_	37.2 (32.7–40.7)	34.7 (31.9–40.0)	38.4 (32.7–44.8)	0.145
RER	1.21 (1.12–1.30)	1.21 (1.14–1.31)	1.21 (1.12–1.31)	0.346
Medical treatment				
Loop diuretics, *n* (%)	24 (82.8)	10 (71.4)	14 (93.3)	0.119
ACEI or ARB or Sacubitril-valsartan, *n* (%)	28 (96.6)	13 (92.9)	15 (100)	0.292
MRA, *n* (%)	21 (72.4)	10 (71.4)	11 (73.3)	0.909
β-blockers, *n* (%)	28 (96.6)	14 (100)	14 (93.3)	0.326

Continuous variables are presented as median (interquartile range) or mean ± standard deviation. Categorical variables are presented as absolute numbers and percentages. ACEI, angiotensin-converting enzyme inhibitor; ARB, angiotensin receptor blocker; CA125, antigen carbohydrate 125; COPD, chronic obstructive pulmonary disease; CPET, cardiopulmonary exercise testing; eGFR, estimated glomerular filtration rate; IHD, ischemic heart disease; FGF23, fibroblast growth factor 23; LVEF, left ventricular ejection fraction assessed by Simpson biplane method; MRA, mineralocorticoid receptor antagonists; NYHA, New York Heart Association functional class; NT- proBNP, the natural logarithm of N-terminal prohormone of brain natriuretic peptide; PeakVO_2_, peak oxygen uptake; RER, respiratory exchange ratio; VE/VCO_2_slope, ventilatory efficiency.

### *Post hoc* power calculation

The estimated statistical power for the given sample size and effect size (predicted marginal mean difference of 0.0264) was 0.588.

### One-month changes in klotho and FGF-23

#### Within-group analysis

**Figure 1:**
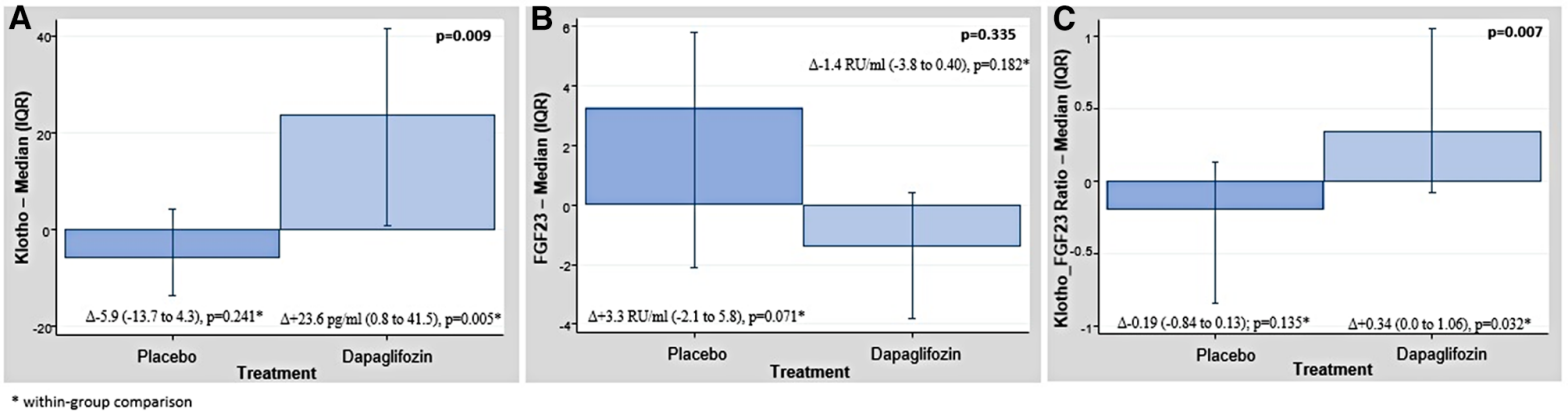
(**A**) Raw data. Changes in the median of klotho across treatment arms. (**B**) Raw data. Changes in the median of FGF-23 across treatment arms. (**C**) Raw data. Changes in the median of klotho/FGF-23 ratio across treatment arms. Values are presented as median (percentile 25% to percentile 75%). FGF-23, fibroblast growth factor 23.

Pre-post comparisons showed that the median (p25%–p75%) of klotho increased in patients allocated to dapagliflozin [Δ+23.6 pg/ml (0.8–41.5), *p* = 0.005]. Compared to baseline, median klotho did not change in patients on placebo [Δ−5.9 (−13.7 to 4.3), *p* = 0.241]. For FGF-23, pre-post analysis did not reveal significant changes in dapagliflozin-arm [Δ−1.4 RU/ml (−3.8 to 0.40), *p* = 0.182] or placebo-arm [Δ+3.3 RU/ml (−2.1 to 5.8), *p* = 0.071]. In those on dapagliflozin, the ratio klotho/FGF-23 significantly increased [Δ+0.34 (0.0–1.06), *p* = 0.032]. We did not find changes in the ratio in those on placebo [Δ−0.19 (−0.84 to 0.13); *p* = 0.135]. Within-group comparisons are shown in Figure 1.

#### Between-group analysis

Compared to placebo, patients on dapagliflozin showed a significant median increase of klotho [Δ+29.5, (12.9–37.2); *p* = 0.009] and a non-significant decrease of FGF-23 [Δ−4.6, (−1.7 to −5.4); *p* = 0.051] as is shown in the Figures 1A,B, respectively. In those on dapagliflozin, the median of the ratio klotho/FGF-23 significantly increased (*p* = 0.007, Figure 1C).

**Figure 2:**
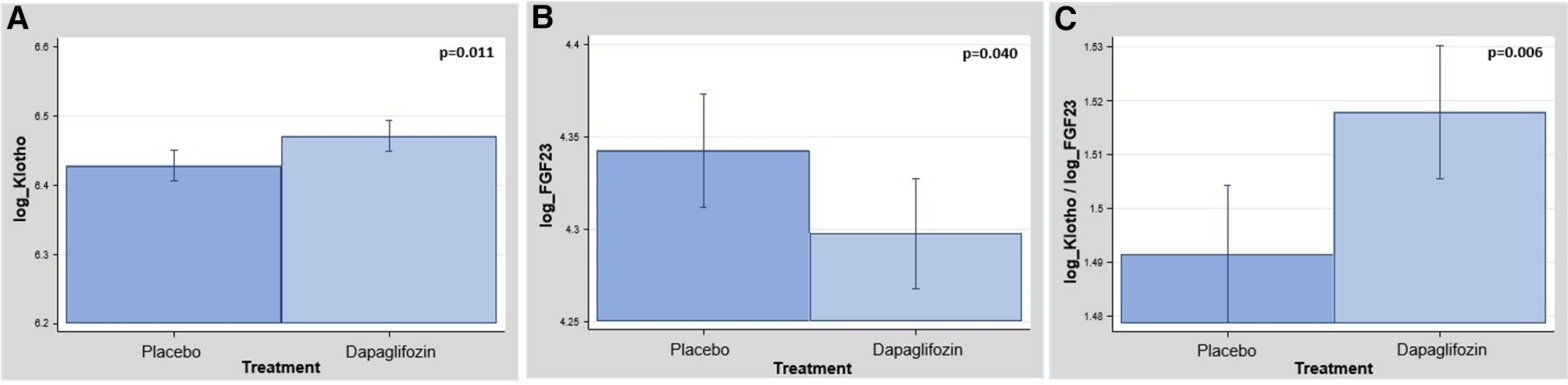
(**A**) Between-treatment changes in logKlotho at 1-month. logKlotho, logarithm of klotho. (**B**) Between-treatment changes in logFGF-23 at 1-month. logFGF-23, logarithm of fibroblast growth factor 23. (**C**) Between-treatment changes in the ratio logKlotho and logFGF-23 at 1-month. logFGF-23, logarithm of fibroblast growth factor 23.

Inferential analyses, adjusting for age, sex, and estimated glomerular filtration rate, confirmed the differences. At 1-month, the logarithm of klotho was higher in patients on treatment with dapagliflozin [Δ+0.04, (CI 95% 0.01–0.07; *p* = 0.011)] as is shown in Figure 2A. Likewise, those patients allocated to dapagliflozin showed lower values of the logarithm of FGF23 [Δ−0.04, (CI 95% −0.09 to −0.01; *p* = 0.040)] as is shown in Figure 2B. When the logarithm of the Klotho/FGF-23 ratio was examined, we also found a highly significant increase in patients allocated to dapagliflozin (Figure 2C).

### Klotho and FGF23 at baseline and changes peakVO_2_

**Figure 3:**
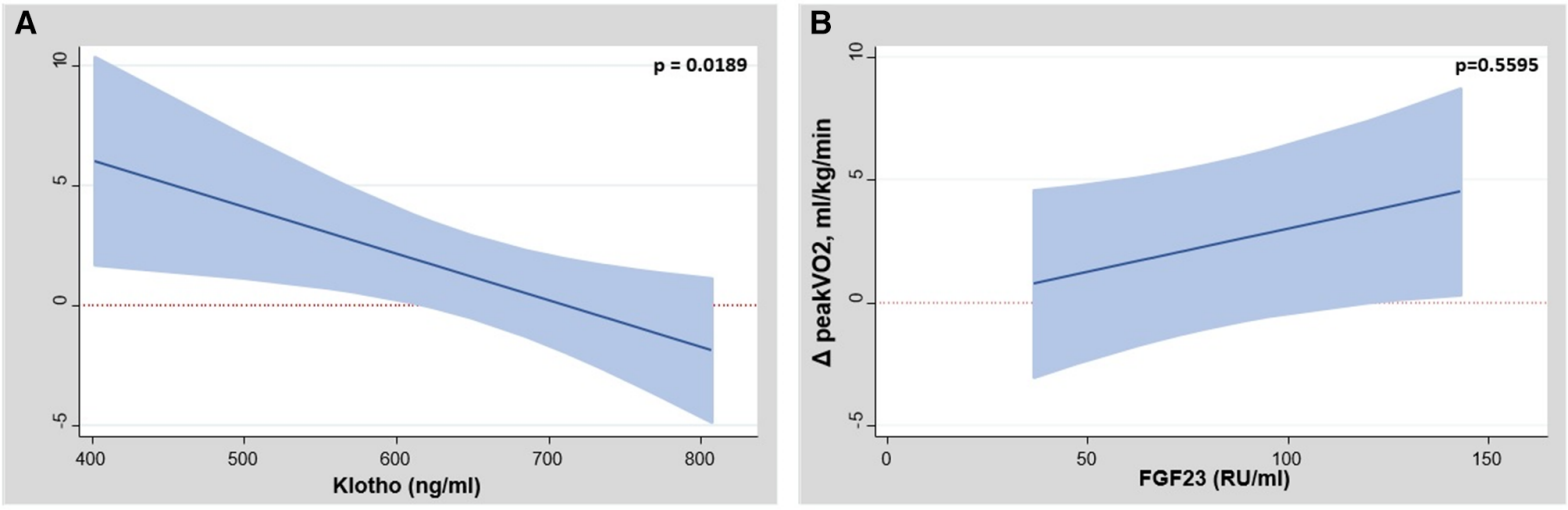
(**A**) Between-treatment changes in peakVO_2_ across baseline values of klotho. peakVO_2_, peak oxygen consumption. (**B**) Between-treatment changes in peakVO_2_ across baseline values of FGF-23. FGF-23, fibroblast growth factor 23; peak VO_2_, peak oxygen consumption.

In this subset of patients, dapagliflozin was associated with a significant improvement in 1-month-peakVO_2_ [Δ+1.02 ml/kg/min (CI 95%: 0.36–1.68; *p* = 0.003)]. Linear regression analysis showed that the lower baseline values of klotho identified those patients with greater benefits in terms of short-term improvement in peak VO_2_ (Figure 3A). We could not find a significant association between baseline FGF-23 and 1-month changes in peak VO_2_ (Figure 3B).

## Discussion

In this *post hoc* analysis of a randomized clinical trial, despite having a low relative statistical power, we found that dapagliflozin treatment in patients with stable HFrEF was associated with a significant increase in klotho at 1 month. Most of the patients included in this study were on stable NYHA II, showed a prior history of hypertension, and were on optimal medical therapy. Interestingly, patients with lower baseline klotho values identified those patients with a greater short-term maximal functional capacity improvement following treatment with dapagliflozin. We could not find a significant decrease in FGF-23 in those on dapagliflozin with the observed data. However, after controlling for potential confounders, there was a signal indicating a short-term reduction of FGF-23.

### FGF-23/klotho axis in HF

FGF-23 is a hormone involved in regulating mineral bone metabolism and maintaining phosphate balance by inhibiting vitamin D synthesis (2). Some studies have revealed that FGF-23 directly impacts the heart, and high levels of FGF-23 in the blood have been linked to adverse cardiovascular outcomes (2). FGF-23 is a hormone involved in regulating mineral bone metabolism and maintaining phosphate balance by inhibiting vitamin D synthesis (2). Additionally, other abnormalities with potential prognostic implications in mineral metabolism are frequent in patients with CV disease. For instance, in patients with stable coronary heart disease, low calcidiol plasma levels predict adverse prognosis in the presence of high FGF-23 levels (4). In patients with established chronic and acute HF, it is associated with disease severity, decreased functional capacity (5), and adverse outcomes (6, 7). The evidence endorsing the role of klotho, an essential coreceptor for FGF-23, in HF is scarcer. Recent studies found that serum klotho concentration was consistently and negatively associated with the presence of HF among US middle-aged and older adults (8). Other studies have found conflicting results, especially in those with chronic kidney disease (9, 10). Although experimental evidence has suggested FGF-23/Klotho axis is involved in left ventricular remodeling and sodium resorption (2), the contribution of this pathway in patients with HFrEF remains inconclusive.

### Effects of SGLT2i in FGF-23 and klotho

To the best of our knowledge, this is the first study reporting a significant modification in the FGF-23/Klotho axis after the initiation of dapagliflozin, correlating with the magnitude of short-term functional response. The mechanisms behind the beneficial effects of SGLT2i in patients with HF seem multiple and not fully understood (1). However, there is some experimental evidence suggesting that SGLT2i has an impact on the FGF-23-klotho axis. For instance, a recent study by Mora-Fernández et al. demonstrated that the association of SGTL2i to diabetic patients in monotherapy with metformin increased the availability of klotho and preserved its synthesis through direct and indirect mechanisms in renal tubular cells, the main site of klotho expression in the body (11). These authors found that SGLT2i decreased albuminuria and urinary TNF-α were inversely associated with changes in urinary Klotho (11). Whether modifications in the FGF-23/Klotho axis play a causal role or merely reflect more complex and profound physiological changes induced by SGLT2i requires further investigation. However, we speculate that increasing klotho could reflect proximal tubule function improvement mediated by decreasing the oxidative stress and inflammatory status of the tubule (12, 13). This increase in klotho could potentially lead to decreased resistance to FGF-23. Moreover, neurohormonal inhibition and improvement in cardiac functional status may also play a role (1, 3). Disarrangement in klotho-FGF-23 axis may identify those patients with greater proximal tubular function impairment, which could, at least in part, explain why the greater short-term improvement in peak VO_2_ was found in those patients with lower baseline klotho levels. More studies are warranted to confirm the current findings in this and other clinical scenarios. Additionally, new studies exploring the mechanisms through which SGLT2i modify the FGF-23 klotho-axis are welcome.

### Study limitations

Some limitations need to be acknowledged. First, this is a *post hoc* analysis of a small subset of patients included in a randomized clinical trial. Second, the power of this study was relatively low. Third, we cannot extrapolate these findings to other clinical scenarios different from stable HFrEF or other SGLT2i different from dapagliflozin. Fourth, with the current data, we cannot unravel the pathophysiological mechanism behind these findings. Fifth, we did not measure microalbuminuria and parameters or phosphocalcium metabolism.

## Conclusions

In patients with stable HFrEF, dapagliflozin led to a significant short-term increase of klotho. Additionally, there were signals of reduction in FGF-23. Lower values of Klotho identified a subset of patients with a greater maximal functional response following treatment with dapagliflozin.

## Data Availability

The raw data supporting the conclusions of this article will be made available by the authors, without undue reservation.

## DAPA-VO_2_ investigators

Patricia Palau, Martina Amiguet, Eloy Domínguez, Clara Sastre, Anna Mollar, Julia Seller, Jose Manuel Garcia Pinilla, Ainoha Larumbe, Alfonso Valle, Juan Jose Gómez Doblas, Rafael de la Espriella, Gema Miñana, Ainhoa Robles Mezcua, Enrique Santas, Vicent Bodí, Juan Sanchis, Domingo Pascual-Figal, Jose Luis Górriz, Antonio Baýes-Genís, Jose Civera, Adriana Conesa, Rim Zakarne, Clara Jiménez Rubio, Alejandro I. Pérez Cabeza, Arancha Díaz Expósito, José David Martínez Carmona, Manuel Luna Morales, Francisco J. Zafra Sánchez, Ángel Montiel Trujillo, Herminio Morillas Climent, and Julio Núñez.
